# Correction: Agundis-Tinajero et al. Deep Learning-Based Automatic Segmentation and Analysis of Mitochondrial Damage by Zika Virus and SARS-CoV-2. *Viruses* 2025, *17*, 1272

**DOI:** 10.3390/v17111520

**Published:** 2025-11-20

**Authors:** Brianda Alexia Agundis-Tinajero, Miguel Ángel Coronado-Ipiña, Ignacio Lara-Hernández, Rodrigo Aparicio-Antonio, Anita Aguirre-Barbosa, Gisela Barrera-Badillo, Nidia Aréchiga-Ceballos, Irma López-Martínez, Claudia G Castillo, Vanessa Labrada-Martagón, Mauricio Comas-García, Aldo Rodrigo Mejía-Rodríguez

**Affiliations:** 1 Department of Sciences, Autonomous University of San Luis Potosí (UASLP), San Luis Potosí 78295, Mexico; 2 Research Center for Health Sciences and Biomedicine, Autonomous University of San Luis Potosí (UASLP), San Luis Potosí 78210, Mexico; 3 Institute of Epidemiological Diagnosis and Reference, InDRE, Mexico City 01480, Mexico; 4 School of Medicine, Coordination for the Innovation and Application of Science and Technology, Autonomous University of San Luis Potosí (UASLP), San Luis Potosí 78210, Mexico

In the original publication [1], Figure 1b was previously published by the authors in the PLoS One, but the citation (reference 14) was inadvertently omitted. The corrected figure legend is provided below:

“**Figure 1.** Thin-section TEM Image. (**a**) Example of an image not used to train the network due to the variety of organelles observed; (**b**) representative example of an image used for network training [14].”.

In the Acknowledgments section, the authors forgot to acknowledge Edson I. Rubio Hernández for his help in generating the initial cultured cells and ZIKV infections. The correct Acknowledgements section is as follows:

“B.A.A.-T. (CVU: 1177797) acknowledges the support of SECIHTI through the Ph.D. fellowship 4038933. M.Á.C.I. (CVU: 1232434) acknowledges the support of SECIHTI through the Ph.D. fellowship 4044561. M.C.-G. thanks SECIHTI for the grant CBF2023-2024-1125 and COPOCYT for the grant 2024-03-M07. The staff of InDRE gratefully acknowledges the technical support provided by Leticia de Jesús Santiago Cruz and Patricia Stephany Justo Berrueta. We also thank Edson I. Rubio-Hernández for his help in generating the initial cultured cells and ZIKV infections, which were published in [14].”

The authors state that the scientific conclusions are unaffected. This correction was approved by the Academic Editor. The original publication has also been updated.
